# Possible roles of genetic variations in chemotherapy related cardiotoxicity in pediatric acute lymphoblastic leukemia and osteosarcoma

**DOI:** 10.1186/s12885-018-4629-6

**Published:** 2018-07-03

**Authors:** Judit C. Sági, Bálint Egyed, Andrea Kelemen, Nóra Kutszegi, Márta Hegyi, András Gézsi, Martina Ayaka Herlitschke, Andrea Rzepiel, Lili E. Fodor, Gábor Ottóffy, Gábor T. Kovács, Dániel J. Erdélyi, Csaba Szalai, Ágnes F. Semsei

**Affiliations:** 1 Department of Genetics, Cell- and Immunobiology, Semmelweis University, 1089 Nagyvárad tér 4., 6 em, Budapest, 611 Hungary; 2 Second Department of Pediatrics, Semmelweis University, Tűzoltó utca 7-9, Budapest, H-1094 Hungary; 3 Central Laboratory, Heim Pal Children Hospital, Üllői út 86, Budapest, H-1089 Hungary; 4 Department of Pediatrics, Oncohaematology Division, Pécs University, József Attila út 7, Pécs, H-7623 Hungary

**Keywords:** Anthracycline, Cardiotoxicity, Cancer, Genetic polymorphisms, Childhood cancer

## Abstract

**Background:**

The treatment of acute lymphoblastic leukemia (ALL) and osteosarcoma (OSC) is very effective: the vast majority of patients recover and survive for decades. However, they still need to face serious adverse effects of chemotherapy. One of these is cardiotoxicity which may lead to progressive heart failure in the long term. Cardiotoxicity is contributed mainly to the use of anthracyclines and might have genetic risk factors. Our goal was to test the association between left ventricular function and genetic variations of candidate genes.

**Methods:**

Echocardiography data from medical records of 622 pediatric ALL and 39 OSC patients were collected from the period 1989–2015. Fractional shortening (FS) and ejection fraction (EF) were determined, 70 single nucleotide polymorphisms (SNPs) in 26 genes were genotyped. Multivariate logistic regression and multi-adjusted general linear model were performed to investigate the influence of genetic polymorphisms on the left ventricular parameters. Bayesian network based Bayesian multilevel analysis of relevance (BN-BMLA) method was applied to test for the potential interaction of the studied cofactors and SNPs.

**Results:**

Our results indicate that variations in *ABCC2*, *CYP3A5, NQO1, SLC22A6* and *SLC28A3* genes might influence the left ventricular parameters. *CYP3A5* rs4646450 TT was 17% among ALL cases with FS lower than 28, and 3% in ALL patients without pathological FS (*p* = 5.60E-03; OR = 6.94 (1.76–27.39)). *SLC28A3* rs7853758 AA was 12% in ALL cases population, while only 1% among controls (*p* = 6.50E-03; OR = 11.56 (1.98–67.45)). Patients with *ABCC2* rs3740066 GG genotype had lower FS during the acute phase of therapy and 5–10 years after treatment (*p* = 7.38E-03, *p* = 7.11E-04, respectively). *NQO1* rs1043470 rare T allele was associated with lower left ventricular function in the acute phase and 5–10 years after the diagnosis (*p* = 4.28E-03 and 5.82E-03, respectively), and *SLC22A6* gene rs6591722 AA genotype was associated with lower mean FS (*p* = 1.71E-03), 5–10 years after the diagnosis.

**Conclusions:**

Genetic variants in transporters and metabolic enzymes might modulate the individual risk to cardiac toxicity after chemotherapy.

**Electronic supplementary material:**

The online version of this article (10.1186/s12885-018-4629-6) contains supplementary material, which is available to authorized users.

## Background

Acute lymphoblastic leukemia (ALL) and osteosarcoma (OSC) occur predominantly in pediatric patients. ALL is the most common childhood hematological malignancy; about 25–30% of childhood cancers are acute leukemia, 80% of which are ALL [1]. Osteosarcoma is a rare bone disease that affects 3–4 people per million and represents 3% of pediatric tumors [2]. Nowadays, the treatment of pediatric ALL is very effective: the majority of patients are cured for the long term. The 5-year event-free survival rate (EFS) is around 80% for ALL and 60% for osteosarcoma patients [2–6].

Unfortunately, despite the use of the indeed efficacious chemotherapeutic drugs, patients have to face serious side effects. Therefore, the primary goal of the scientific research is now not only to increase the survival rate, but to identify and reduce the acute and late toxic side effects of chemotherapy and to improve the quality of life in adulthood [4, 7–10]. The risk for developing health problems is increased 8-fold in pediatric cancer survivors within 30–40 years after diagnosis compared to their siblings; 50% of the sibs experience severe, disabling or life-threatening events, including death by the age of 50. One of the late toxic side effects of chemotherapy in childhood ALL is cardiotoxicity [5, 7, 11, 12]. A 30-year-old survivor might face treatment-related cardiac damage usually characteristic for much older patients [13–15]. There is a need for preventing cardiac damage especially in children, because they can live for decades after treatment [16]. Several treatment regimens introduce dose reduction in some cases to decrease late side effects, but childhood cancer survivors still require long-term follow-up for their prevention and treatment. The constant monitoring of patients is important in order to identify subclinical anomalies before the clinical symptoms occur [17–20].

Anthracyclines are among the most essential and highly effective chemotherapeutic agents in the treatment of both hematological malignancies and solid tumors (e.g. leukemia, lymphoma, breast cancer, and sarcoma) [21–25], and belong to the backbone of childhood ALL and osteosarcoma treatment protocols all around the globe [4]. However, anthracyclines damage cardiomyocytes which can manifest during the therapy, years or even decades after the exposure to chemotherapeutic agents [18, 26–30]. The pathophysiology of the toxicity is not completely understood, but it is likely that both the drug and its metabolites are cardiotoxic [31, 32]. Despite the fact that anthracycline-induced cardiotoxicity (ACT) is well known, the toxicity is unpredictable [33–35]. Rates of cardiotoxicity increase the higher the cumulative dose, with doses above 500 mg/m2 resulting in unacceptable rates of cardiac toxicity; therefore, most of the treatment protocols limit the use of these drugs below this value [18, 36]. However, there are patients with cardiac problems who received very low doses of anthracyclines while others were administered with high doses and escaped the side effect. The variable development of anthracycline cardiotoxicity suggests that the genetic background of the patients is important in this side effect [37–45].

The comparability of pharmacogenomics research results is hampered by the heterogeneity of the populations under study, applied treatment protocols and investigated parameters. Half of the studies were executed on former pediatric patient populations and there is an urgent pressure to translate these research evidences into clinical practice [46–50]. For this purpose one of the newest publications of the topic contains evidence-based clinical practice recommendations for pharmacogenomic testing. They emphasize *RARG* (Retinoic Acid Receptor Gamma) rs2229774, *SLC28A3* (Solute Carrier Family 28 Member 3) rs7853758 and *UGT1A6* (UDP Glucuronosyltransferase Family 1 Member A6) rs17863783 as genetic variants which have the strongest association with ACT [49]. SNPs in the genes of anthracycline transporters *ABCB1* (ATP Binding Cassette Subfamily B Member 1) and *ABCC1* (ATP Binding Cassette Subfamily C Member 1) also associated with ACT in anthracycline-treated children [37]. *SLC22A7* (Solute Carrier Family 22 Member 7) and *SLC22A17* (Solute Carrier Family 22 Member 17) were also in connection with cardiotoxicity among patients with osteosarcoma [2]. Studying adult patients with non-Hodgkin lymphoma Wojnowski et al. found associations between cardiotoxicity and SNPs in the NAD(P)H oxidase complex, and *ABCC1* and *ABCC2* (ATP Binding Cassette Subfamily C Member 2) transporters [38]. The function of cytochrome P450 enzymes is crucial in the metabolism of several drugs, among their coding genes e.g.: *CYP3A5* (Cytochrome P450 Family 3 Subfamily A Member 5) had importance in this context. In a genome wide association study *RARG* has been identified as a promising gene [48].

These results support the hypothesis that the genetic features of the patients may influence chemotherapy-related cardiotoxicity. However, further independent studies are needed to confirm these findings. From the scientific literature and databases we selected 70 SNPs in 24 candidate genes coding for xenobiotic transporters and metabolizing enzymes, and searched for associations between these genetic variations, and acute or late left ventricular damage in pediatric acute lymphoblastic leukemia and osteosarcoma patients.

## Methods

### Patients

In this study, patients with pediatric acute lymphoblastic leukemia (ALL) or osteosarcoma (OSC), aged 0–18 years at diagnosis, were enrolled retrospectively (*n* = 680). Children with ALL had undergone chemotherapy between 1989 and 2015 in 6 Hungarian pediatric oncology centers, patients with OSC were treated between 1989 and 2015 at the Second Department of Pediatrics, Semmelweis University. Patients were excluded from the analysis because of Down syndrome (*n* = 7), previous cardiac problems or any concomitant disease with potential cardiac complications (adrenoleukodystrophy, agenesia renis, cardiac arrhythmia, congenital hypothyroidism, cystic fibrosis, ventricular septal defect, VACTERL) (*n* = 12). Detailed description of the ALL (*n* = 622) and OSC (*n* = 39) patients included in the statistical analysis is shown in Table 1.

**Table 1:** Characteristics of the studied populations

	Patients with ALL	Patients with OSC	Total
Number of patients	622	39	661
Gender *n* (%)			
Male	372 (60)	27 (69)	399 (60)
Female	250 (40)	12 (31)	262 (40)
Age at diagnosis (%)			
< 1 yr. *n*	7 (1)	0	7 (1)
1–10 yr. *n*	505 (81)	9 (23)	515 (78)
> 10 yr. *n*	109 (18)	30 (77)	138 (21)
Mean ± SD *yr*	6.39 (±4.3)	13.1 (±3.5)	6.6 (±4.3)
Median (range) *yr*	5.2 (0–18)	13.2 (5–18)	5.3 (0–18)
Risk group *n* (%)			
SR	165 (27)	3 (7)	168 (25)
IR	355 (57)	24 (62)	379 (58)
HR	100 (16)	12 (31)	112 (17)
Chemotherapy protocol *n* (%)			
Protocols before 2000^1^	325 (52)	−	325 (52)
Protocols after 2000^2^	297 (48)	−	297 (48)
OSC protocols	−	39	39
Anthracycline dose^3^ (range, mg/m^2^)	60–840	180–360	60–840
Anthracycline dose *n* (%)			
≤ 240 mg/m^2^	457 (74)	6 (15)	463 (70)
> 240 mg/m^2^	163 (26)	33 (85)	196 (30)
Patients with pathological FS^4^ *n*	18	2	20

Data are reported as numbers with percentages, unless mentioned otherwise. Abbreviations: ALL, acute lymphoid leukemia; OSC, osteosarcoma; SD, standard deviation; SR, standard-risk; IR, intermediate-risk; HR, high-risk; FS, left ventricular fractional shortening. ^1^ALL patients treated with ALL BFM 88, ALL BFM 90, ALL BFM 95, Interfant 98, NHL BFM 90 or NHL BFM 95 protocol. ^2^ALL patients treated with ALL IC BFM 2002, ALL IC BFM 2009 or Interfant 2006 protocol. ^3^Cumulative anthracycline dose in doxorubicin or daunorubicin equivalent doses during the treatment according to protocol. ^4^FS below 28%

Patients with ALL were treated according to one of the following study protocols: ALL BFM (Berlin–Frankfurt–Münster) 88, ALL BFM 90, ALL BFM 95, ALL IC-BFM (ALL Intercontinental) 2002 or ALL IC-BFM 2009; Interfant 98 or Interfant 2006. The chemotherapy regimen was described in detail in our previous article [51]. Patients diagnosed with ALL and OSC were treated with anthracyclines in the first year of chemotherapy. The chemotherapy protocols differed slightly in the number or dosage of anthracycline-administrations. In the low-risk and medium-risk groups of patients with ALL the cumulative anthracycline (i.e. doxorubicin equivalent) doses were between 180 and 240 mg/m^2^; in the high-risk group and in the therapy of relapsed patients it were between 240 and 380 mg/m^2^. The anthracycline treatment of patients with osteosarcoma was based on the COSS (German-Austrian-Swiss osteosarcoma study group) -86 and COSS-96 protocols. Treatment of patients with OSC included cumulative doxorubicin doses 360 mg/m^2^ for standard-risk group patients or 180 mg/m^2^ for the high-risk group. For detailed description of the used COSS based protocols see Hegyi et al., 2016 [52]. In our cohort 29% of the patients received 12 Gy cranial radiotherapy according to the schedule of the ongoing BFM protocol (ALL BFM 90 or ALL BFM 95 protocols in 22% of the patients).

Informed consent was requested from legal guardians of the patients or from the participants above the age of 16 (6% of patients). The study was approved by the Ethics Committee of the Hungarian Medical Research Council and conducted according to the principles of the Declaration of Helsinki.

The patients were followed-up by echocardiography (ECHO) routinely in the clinical practice to monitor their left ventricular function. All ECHOs were performed by the pediatric cardiologists in the Hungarian pediatric oncology centers. Left ventricular end-diastolic-diameter (LVEDD) and left ventricular end-systolic diameter (LVESD) data were collected from the patients’ medical records. Left ventricular ejection fraction (EF) and left ventricular fractional shortening (FS) were determined: EF = (LVEDD^3^-LVESD^3^)/LVEDD^3^; FS = (LVEDD-LVESD)/LVEDD. Measurements were performed before the initiation of therapy, several times during the treatment and annually after finishing treatment. FS and EF data were analyzed in follow-up categories, which are: 1) at the diagnosis (used as a control); 2) in acute phase: during the intensive chemotherapy phase; 3) during oral maintenance chemotherapy; 4) at the end of the treatment, which is after the oral maintenance chemotherapy period completed 2 or 3 years after the diagnosis; 5) from the end of the treatment until 5 years after the diagnosis; 6) 5–10 years after the diagnosis; 7) 10–15 years after the diagnosis; 8) more than 15 years after the diagnosis. For detailed description of the follow-up categories see Table 2. Not all of the ECHO records were available, because of the retrospective data collection. Only the latest ECHO of each patient was used in each follow-up category.

**Table 2:** Follow-up categories with echocardiography parameters

Follow-up category	Patients with ALL	Patients with OSC	Total population		
	N (mean FS ± SD)	N (mean FS ± SD)	N (mean FS ± SD)	Decreased not decreased FS, N^1^	OR (95% CI) ^2^
At the diagnosis	358	29	387		
	41.5 ± 6.1	39.6 ± 4.4	41.4 ± 6.0		
< 1 yr. from diagnosis^1^	275	5	280	104 | 83	1.0
	40.4 ± 6.1	40.2 ± 5.3	40.4 ± 6.1		
1–2 yr. from diagnosis	46	3	49	19 | 10	1.5 (0.6–3.4)
	41.4 ± 6.0	39.9 ± 2.3	41.3 ± 5.9		
End of the treatment	287	28	315	105 | 98	0.9 (0.6–1.3)
	40.0 ± 5.6	38.4 ± 6.3	39.9 ± 5.7		
2–5 yr. from diagnosis	229	35	264	77 | 73	0.8 (0.5–1.3)
	40.4 ± 5.7	38.1 ± 5.2	40.1 ± 5.7		
5–10 yr. from diagnosis	265	36	301	70 | 76	0.7 (0.5–1.1)
	40.1 ± 5.5	40.3 ± 5.6	40.1 ± 5.5		
10–15 yr. from diagnosis	133	19	152	24 | 36	0.5 (0.3–1.0)
	40.4 ± 5.4	39.9 ± 5.2	40.3 ± 5.3		
> 15 yr. from diagnosis	24	8	32	5 | 3	1.3 (0.3–5.7)
	37.6 ± 7.5	40.7 ± 6.1	38.4 ± 7.2		

The decrease of FS was calculated patient by patient in every category compared to the individual value at diagnosis if these data were available. ^1^ Number of patients with a decreased FS per number of patients with a not decreased FS. ^2^ Compared to the second category. Abbreviations: CI, confidence interval; FS, left ventricular fractional shortening; OR, odds ratio; N, number; SD, standard deviation

The worst heart function of each patient was used to define patients for the case-control type study. Cases were those who had echocardiograms with FS ≤ 28% at any time point during the follow-up (*n* = 20); patients, who received the same chemotherapy but never had FS ≤ 28% were regarded as controls (*n* = 641).

The alteration of FS was computed and analyzed as dichotomous variable, which was defined as the difference between the FS value at diagnosis and at the end of the treatment. In this study, patients with decreased FS (*n* = 105) were compared to those with increased FS (*n* = 94). Difference between the FS value at diagnosis and at the last follow-up time point was also computed, 170 patients with decreased FS were compared to those with increased FS (*n* = 152).

### Laboratory methods

Peripheral blood samples were taken from children with ALL in remission. DNA was isolated from blood using Qiagen isolation kits according to the manufacturer’s instructions (QIAmp DNA Blood Midi or Maxi Kit, Qiagen, Hilden, Germany).

Based on the scientific literature, 70 single nucleotide polymorphisms (SNPs) in 26 genes were selected and genotyped. These genes encode transporters involved in drug import or elimination as well as enzymes in the metabolism of the chemotherapeutic agents. Candidate genes were chosen from previous candidate gene studies in this field if the gene or gene family were found to be associated with cardiotoxicity in more than two studies (ABCB1, ABCC1, ABCC2, GSTP1, SLC22A17, SLC22A6, SLC22A7, SLC22A8, SLC28A3) [48] or were found in relation to cardiotoxicity in large-scale or genome-wide association studies (BCL2, HAS3, RARG) [53–55]. Previously not validated potential candidate genes were also selected if those were important in the transport or metabolism of cardiotoxic drugs used in chemotherapy (AKR1A1, AKR1C3, ABCG2, CEP72, CYP3A4, CYP3A5, NQO1, NQO2, MTHFR) [46]. SNPs were selected prioritized on the basis of their estimated functionality in this order: non-synonymous SNPs, SNPs in the promoter and the 3’-UTR (3′-untranslated region) region, synonymous SNPs and intronic SNPs. During the selection the minor allele frequency data of the SNPs were validated using HapMap database release No. 27 and the CEU population (CEPH: Utah residents with ancestry from northern and western Europe) [56]. Information on the selected SNPs is shown in an additional table file in more detail [see Additional file 1]. Genotyping 63 of the SNPs was conducted using TaqMan® OpenArray™ Genotyping System (Thermo Fisher Scientific, Waltham, MA, USA) following the manufacturer’s instructions at the Department of Medical Chemistry, Molecular Biology and Pathobiochemistry, Semmelweis University (Budapest, Hungary). Detailed description of this procedure can be found in the article of Banlaki et al. [57]. Other 7 SNPs were genotyped using KASPar (KBioscience Competitive Allele-Specific Polymerase chain reaction)-on-Demand prevalidated assays (LGC Genomics, Berlin, Germany) on 7900HT Fast Real-Time PCR System (Thermo Fisher Scientific Waltham, MA, USA). The genotyping was unsuccessful in the case of three SNPs; call rate for the other SNPs was higher than 87.5%.

### Statistical analysis

Allele frequencies were tested by allele counting, HWE (Hardy-Weinberg equilibrium) was studied using an on-line software [58], significant violation of HWE was considered where *p* ≤ 8.90E-03. In our case-control and follow-up studies, univariate and multivariate logistic regression and multi-adjusted general linear model were performed to investigate the influence of genetic polymorphisms on the left ventricular parameters. The analyses were adjusted for potential confounders, which were age at the time of diagnosis (years), gender (male-female), chemotherapy protocols (before 2000, after 2000 and OSC protocols; also reflects radiotherapy), risk groups (standard, intermediate, high-risk) and cumulative dose of anthracycline (≤ or > 240 mg/m^2^). The analyses were performed studying the genotypes separately (11 vs. 12 vs. 22), using recessive (11/12 vs. 22) or dominant (11 vs. 12/22) models, with the common homozygotes signed as 11. EF and FS is indicated in the text with the standard error (SE) of the estimate of the mean. In order to deal with multiple comparisons the Benjamini-Hochberg false discovery rate (FDR) method with type I error rate of 10% (p ≤ 8.90E-03) was applied as correction (with 201 analyses performed for 67 SNPs and each phenotype) [59, 60]. Analyses and preparation of the figures were performed using IBM SPSS Statistics 23.0 (IBM Corporation, Armonk, NY, USA) and RStudio Version 1.0.136 (RStudio, Boston, MA, USA) programs. Estimated haplotype frequency in cases and controls and the haplotype-specific odds ratio (OR) were calculated by the Haploview 4.1 software [61]. The power of the analyses was calculated at a significance level of 0.05 using SPSS Statistics 23.0 program. Bayesian network based Bayesian multilevel analysis of relevance (BN-BMLA) method was applied to test for potential interaction of the studied cofactors and SNPs. The BN-BMLA was described in our previous article [62].

## Results

In this study, altogether 70 SNPs were genotyped. The minor allele frequencies of the SNPs are presented in an additional table file in more detail [see Additional file 1]. Genotype distributions were in Hardy-Weinberg equilibrium except for one SNP (*AKR1A1* (Aldo-Keto Reductase Family 1 Member A1) rs2934859) which was excluded from the analysis. Genotyping was unsuccessful in the case of three SNPs. Thus, the genotyping results of 66 SNPs were used for the evaluations. Minor allele frequencies in our population were found to be more than 7% for all of the SNPs. The analyses performed on the population had adequate power (≥75%) for all of the results.

### Case-control analysis

The potential roles of genetic variations of candidate genes in the changes of the left ventricular function of children with ALL or OSC after anthracycline therapy were investigated. Ejection fraction (EF) and fractional shortening (FS) were used to monitor the left ventricular function. Patients who had FS ≤ 28% any time during the follow-up were regarded as cases, those who received the same chemotherapy, but never had FS ≤ 28% were regarded as controls. To assess the possible association of the genotypes with cardiotoxicity, the genotype and allele frequencies in the two groups were compared.

**Fig. 1:**
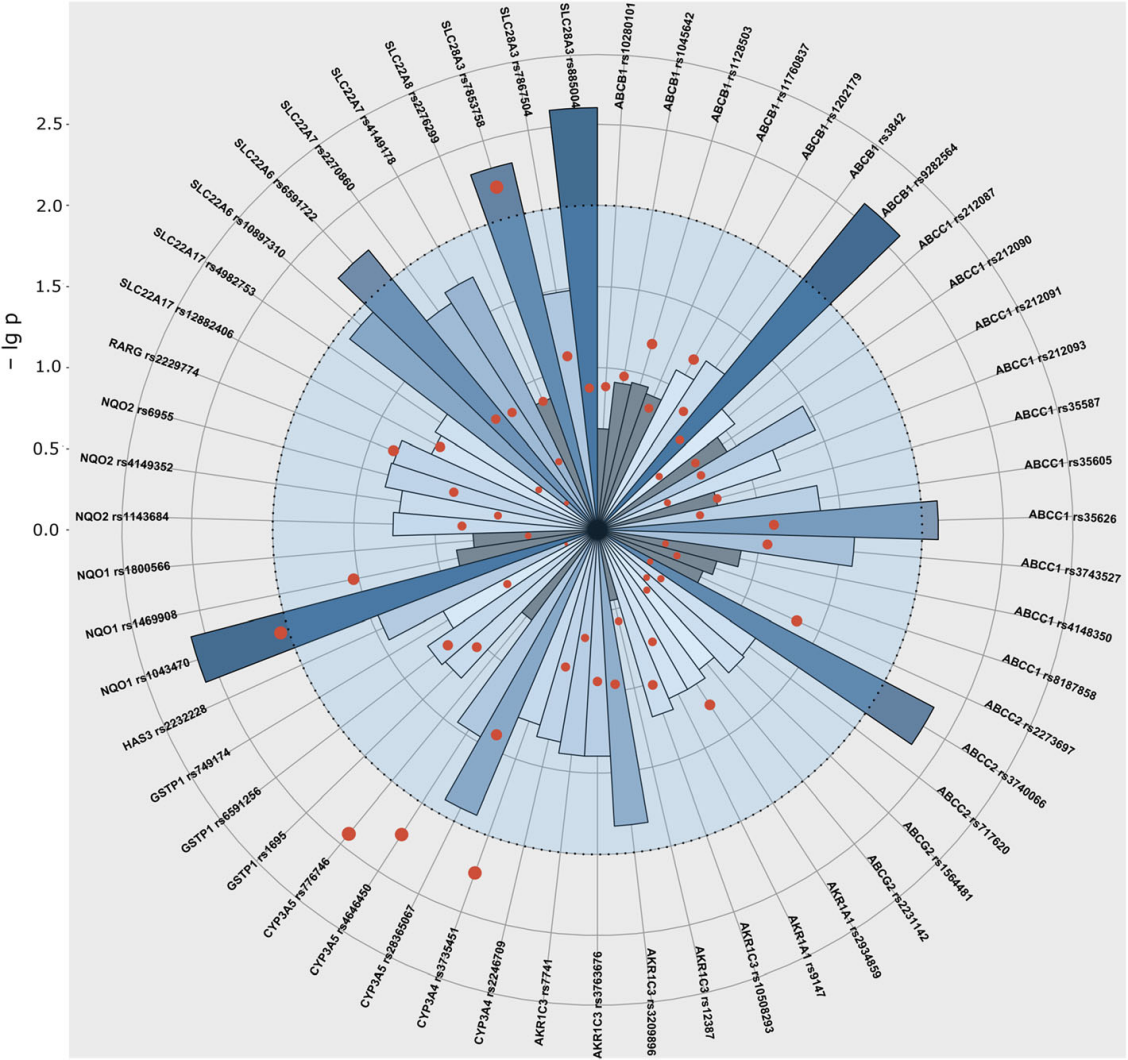
The *p* values of the follow-up and case-control studies of the ALL population. Results of the analysis including data of ALL patients are presented in this Figure. The p values are illustrated in a polar coordinate system, where the circular grids represent the negative logarithm of p values (axis on the left can be projected to grids). Intermittent line indicates border at *p* = 0.01 (− lg *p* = 2). Columns show the results of the follow-up analysis of FS, darker shades of blue mean stronger significance. The lowest p values were chosen from every follow-up category and from every model, if the number of cases was above 5. (The time of diagnosis was excluded.) Results of the case-control study are shown with red dots, sizes proportional with stronger significance. Most significant results of this plot were studied further on the total cohort including osteosarcoma patients as well

Multi-adjusted logistic regression analyses were used on the full cohort, while univariate logistic regression analyses were implemented on various subpopulations. Case-control analysis was performed for ALL patients in case of all SNPs. Among these, the ones with at least 2 cases in one group are shown in Fig. 1. Risk of pathological FS was significantly influenced by SNPs in *CYP3A5* and *SLC28A3* genes. *CYP3A5* rs4646450 TT was 17% among ALL cases and 3% in ALL patients without pathological FS (*p* = 5.60E-03; OR = 6.94 (1.76–27.39)). *SLC28A3* rs7853758 AA was 12% in ALL cases, while only 1% among controls (*p* = 6.50E-03; OR = 11.56 (1.98–67.45)). These two SNPs were analyzed in the whole population including both ALL and OSC patients.

The genotype distribution of the *CYP3A5* rs4646450 differed significantly between cases and controls in the combined cohort (ALL and OSC patients) (*p* = 4.81E-03; OR = 7.25 (1.83–28.78)). Among cases (*n* = 20) 15% had TT genotype while this value was 2.8% in controls. The genotype distribution of the *SLC28A3* rs7853758 SNP was not different between cases (10.5%) and controls (1.3%) in the combined cohort (*p* = 1.00E-02; OR = 9.837 (1.73–56.02) if considering the corrected *p* value.

**Fig. 2:**
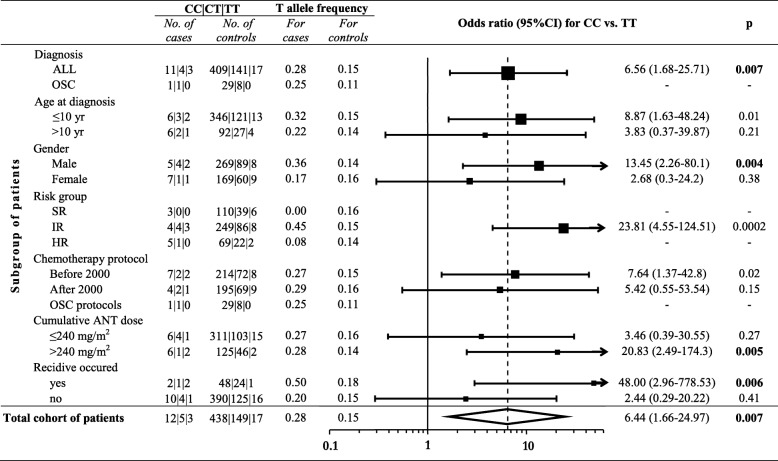
Odds ratios for cardiotoxicity associated with the CYP3A5 rs4646450 genotype among subgroups of patients and also in the whole cohort. Results of the univariate logistic regression analysis performed on subpopulations of patients and also on the total cohort of patients. Subpopulations are determined based on the following factors: diagnosis, age at diagnosis, gender, risk group, chemotherapy protocol, cumulative ANT dose, recidive occurred. Black boxes represent OR, the number of cases is proportional with the width of the boxes. The lengths of the horizontal lines depict the 95% confidence intervals. Analysis of OR was not accomplished if the number of cases was 0. Abbreviations: ALL, acute lymphoblastic leukemia; OSC, osteosarcoma; no, number; yr., year; SR, standard-risk; IR, intermediate-risk; HR, high-risk

Subsequently, it was investigated whether *CYP3A5* rs4646450 was associated with cardiotoxicity in various subpopulations determined by clinical characteristics of the patients (Fig. 2). The *CYP3A5* rs4646450 TT genotype was associated with cardiotoxicity in patients with ALL (*p* = 7.00E-03; OR = 6.56 (1.68–25.71)). Similar association was found when analyzing only male patients (*p* = 4.00E-03; OR = 13.45 (2.26–80.1)) or intermediate-risk patients (*p* = 2.00E-04; OR = 23.34 (4.46–122.07)).

Usage of radiation therapy did not associate with FS reduction below 28% in our cohort. Haplotype analyses were carried out to study the association of haplotype blocks of genes in cardiotoxicity, but no significant results were found.

### Follow-up analysis

All of the SNPs were analyzed in relation with EF an FS in the acute lymphoid leukemia population in every follow-up category with all of the three models using multi-adjusted general linear model. Summary of the results from the analyses is shown in Fig. 1. Results with the lowest p value are depicted, except those with less than 5 patients in one group. Significant results of this analysis are shown in Table 3. SNPs with *p* values < 0.01 were analyzed in the whole population including both ALL and OSC patients.

**Table 3:** Significant results of the follow-up analysis in the acute lymphoid leukemia population

Gene	SNP	Genotype group 1 / group 2	Mean FS % ± SE genotype group 1 (N)	Mean FS % ± SE genotype group 2 (N)	*P* value	Follow-up category
*ABCB1*	rs9282564	AA / AG + GG	41.5 ± 0.7 (100)	37.9 ± 1.1 (29)	2 .50E-03	10–15 years after Dx
*ABCC1*	rs35626	GG / GT + TT	41.0 ± 0.6 (92)	39.0 ± 0.6 (127)	7 .90E-03	2–5 years after Dx
*ABCC2*	rs3740066	GG / GA / AA	39.5 ± 0.5 (112)	40.8 ± 0.5 (112) / 42.9 ± 0.9 (33)	4 .50E-03	5–10 years after Dx
*NQO1*	rs1043470	CC / CT + TT	40.9 ± 0.5 (198)	38.1 ± 0.9 (53)	2 .60E-03	acute phase
*SLC22A6*	rs6591722	TT + TA / AA	40.7 ± 0.4 (227)	37.8 ± 1.0 (28)	5 .90E-03	5–10 years after Dx
*SLC28A3*	rs7853758	GG / GA + AA	41.3 ± 0.7 (96)	38.4 ± 1.1 (36)	4 .80E-03	10–15 years after Dx
*SLC28A3*	rs885004	GG / GA + AA	41.3 ± 0.7 (95)	38.0 ± 1.1 (33)	2 .50E-03	10–15 years after Dx

Results are from multivariate general linear model performed on the ALL cohort adjusted for potential confounders. Abbreviations: Dx, diagnosis; FS, fractional shortening; N, number; SE, standard error

**Fig. 3:**
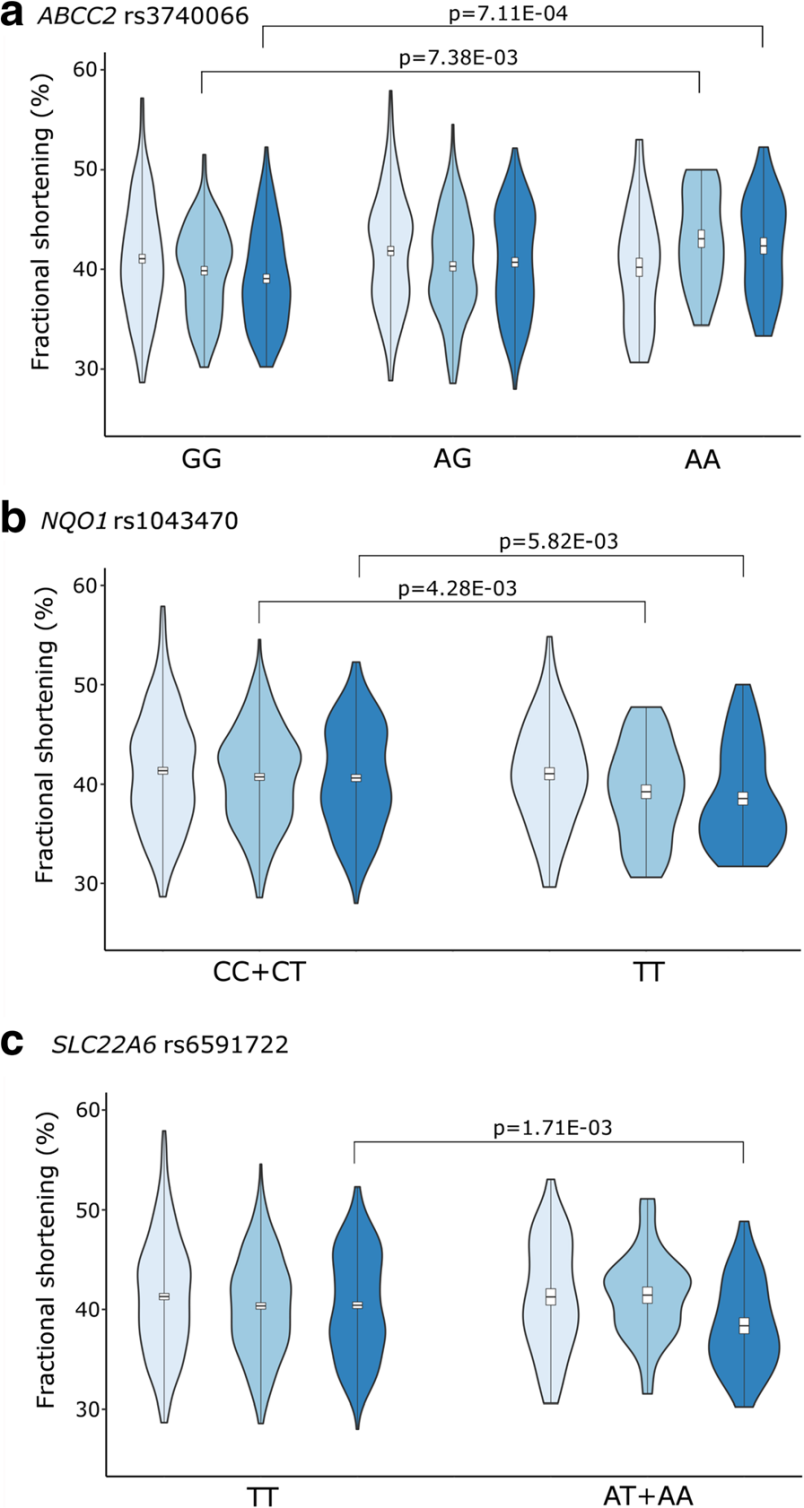
Violin plot of fractional shortening in the total population. FS (%) by genotypes is shown in different follow-up categories. Light blue is the time of diagnosis, medium blue is the time of the anthracycline administration (acute phase), dark blue is the follow-up 5–10 years after therapy. FS is indicated in box plots, box is mean ± S.D., whiskers are means ±3 S.D. Violin plot describes the distribution of FS data, records out of mean ± 3SD are not shown. A: *ABCC2* rs3740066; B: *NQO1* rs1043470; C: *SLC22A6* rs6591722

*ABCC2* rs3740066 common GG genotype was associated with the poorest left ventricular function during the intensive chemotherapy phase (acute phase) in the whole population (Fig. 3). Patients with GG genotype had lower mean FS (39.5% ± 1.06) value, compared to patients with AA genotype (mean FS = 42.9% ± 1.4; *p* = 7.38E-03). The *ABCC2* rs3740066 GG genotype was also associated with significantly lower mean FS (39.1% ± 0.5; *p* = 7.11E-04) at 5–10 years after the diagnosis, whereas higher mean FS rates were related to the other genotypes (40.6% ± 0.5, 42.4% ± 0.8; AG, AA, respectively).

Patients with *NQO1* (NAD(P)H Quinone Dehydrogenase 1) rs1043470 rare T allele had significantly lower mean left ventricular function rates during both phases: in the intensive chemotherapy phase (acute phase) and 5–10 years after the diagnosis (Fig. 3). In the acute phase the T allele was associated with lower mean FS (38.1% ± 1.2; *p* = 4.28E-03), while patients with at least one C allele had FS = 40.7% ± 0.9. Between 5 and 10 years after the therapy *NQO1* rs1043470 rare T allele was associated with lower mean FS (38.5% ± 0.7, *p* = 5.82E-03), while the values represented with the C allele were higher (FS = 40.6% ± 0.4).

*SLC22A6* (Solute Carrier Family 22 Member 6) gene rs6591722 rare AA genotype was associated with lower mean FS (37.5% ± 0.9, *p* = 1.71E-03) 5–10 years after the diagnosis compared to values of TT and TA genotypes (FS: 40.6% ± 0.4) (Fig. 3).

The other SNPs were not associated with heart function parameters. Results regarding ejection fraction were consistent in the direction of those described above with fractional shortening.

### Analysis of fractional shortening alteration

When computing alteration of fractional shortening from diagnosis until the end of the treatment or the last echo, difference of FS was more than 3% or less than 3% in 67–71% of the patients in all groups. The alteration of fractional shortening from diagnosis until the end of the treatment or the last echo ever measured was also analyzed for patients with ALL for all SNPs. Among these the ones with at least 2 cases in one group are shown in Fig. 1, if the *p* value was lower than the p value of case-control analysis. SNPs with *p* values < 0.01 are shown in Table 4. These were analyzed in the whole population including both ALL and OSC patients and were not significant.

**Table 4:** Significant results of the analysis of fractional shortening alteration in the acute lymphoid leukemia population

Gene	SNP	Genotype group 1 / group 2	Patients with decreased FS in genotype groups N (%)	Patients with increased FS in genotype groups N (%)	*P* value	OR (CI 95%)
Alteration of FS: diagnosis vs. end of therapy						
*CYP3A4*	rs3735451	AA / AG + GG	74 (82) / 16 (18)	52 (63) / 31 (37)	5.70E-03	0.36 (0.18–0.74)
*CYP3A5*	rs776746	GG / GA + AA	81 (91) / 8 (9)	60 (73) / 22 (27)	3.80E-03	0.26 (0.11–0.65)
Alteration of FS: diagnosis vs. last echocardiography						
*NQO1*	rs1043470	CC / CT + TT	111 (85) / 41 (15)	112 (73) / 20 (27)	8.90E-03	0.44 (0.24–0.81)

Results are from logistic regression performed on the ALL cohort adjusted for potential confounders. Abbreviations: CI, confidence interval; FS, fractional shortening; N, number; OR, odds ratio

### Bayesian network based Bayesian multilevel analysis of relevance

Bayesian network based Bayesian multilevel analysis of relevance (BN-BMLA) method was performed for SNPs in the *ABCB1, ABCC1, ABCC2, ABCG2, AKR1A1, AKR1C3, CYP3A4, CYP3A5, GSTP1, HAS3, NQO1, NQO2, RARG, SLC22A17, SLC22A6, SLC22A7, SLC22A8 and SLC28A3* genes along with cofactors. This method aims to find the most probably strongly relevant variables with respect to the case-control status of the patients. The strongly relevant variables have a direct influence on the target. Values for posterior probability of strong relevance (P) range from 0 to 1, where *P* = 1 means that the probability of the given variable is 100% relevant with respect to the case-control status. Our analyses revealed potentially strongly relevant effects of a SNP in gene *CYP3A5* (rs776746, *P* = 0.42), two SNPs in gene *NQO1* (rs1043470 and rs1469908, P = 0.42 and 0.34, respectively), two SNPs in gene *SLC28A3* (rs7853758 and rs885004, *P* = 0.55 and 0.36, respectively), and several cofactors (age at the time of diagnosis, *P* = 0.72; gender, *P* = 0.44; risk group, *P* = 0.73; diagnosis (ALL vs. OSC), *P* = 0.8 and cumulative dose of anthracycline, *P* = 0.64). Besides, several interaction effects were found between the variables. Among these, the two SNPs (rs7853758 and rs885004) in gene *SLC28A3* showed the strongest interaction. However, as the number of cases was low, these interaction effects could not be confirmed with logistic regression models using interaction terms.

## Discussion

In this study, we evaluated the association of 66 single nucleotide polymorphisms and anthracycline- induced cardiotoxicity (ACT) developed during or after the treatment in acute lymphoblastic leukemia and osteosarcoma patients. SNPs in four investigated genes (*ABCC2, NQO1, SLC22A6* and *SLC28A3*) were associated with decreased FS and EF. Regarding the aforementioned genes, the acute phase and the period of 5–10 years after the diagnosis were especially important. *CYP3A5* SNP appeared to be a predictor for ACT; the association was more prominent in boys, in ALL patients and in the intermediate risk group.

It must be noted that there are some potential biases of this study. Because of the retrospective data collection not all of the ECHO records were available. Therefore, the analysis of ECHO was not possible for every year; categories of follow-up were generated. Only the data of the latest ECHO of each patient were used in each follow-up category, the redundant echocardiography measurements were excluded. Nevertheless, the large patient population and long follow-up make our study notable. Also, it has to be mentioned that patients who died before the period of sample collection are underrepresented in our cohort. In our opinion, this is not a relevant bias, as late effects only manifested and have relevance in survivors. Furthermore, according to the data of the Hungarian Pediatric Cancer Registry, only three patients in our cohort did die of cardiac-related events (endocarditis, ventricular insufficiency and one patient died of cardiomyopathia). Controls have 1–7 echocardiogram assessments in our cohort (21% of the patients had only one echo and 60% of patients had 3 or more echos). Still we think that our results are real in our cohort, as statistical analyses performed using smaller cohort of controls show the same direction.

### ABCC2

In our study, during the treatment and after 5–10 years of the therapy *ABCC2* rs3740066 common GG genotype was associated with decreased FS and EF values. *ABCC2 (a.k.a. MRP2*; 10q24.2) is a member of the ATP binding cassette subfamily. ABCC2 is responsible for organic anion transmembrane transport and its substrates also include anticancer drugs, antibiotics and statins. The efflux activity of ABCC2 is involved in multidrug resistance. Expression of *ABCC2* is at critical sites of uptake and elimination, including the hepatobiliary tract, intestine, kidney and blood-tissue barriers [63]. *ABCC2* is a frequently investigated gene for instance in drug-related toxicities, in therapy-response, resistance against various drugs, in carcinogenesis and in the outcomes of osteosarcoma and leukemia [64–70]. There are also several findings in the field of cardiotoxicity regarding *ABCC2*. Wojnowski et al. studied acute and chronic ACT in adult patients with Non-Hodgkin lymphoma (NHL). Acute ACT was associated with one haplotype of the *ABCC2* gene (rs8187694-rs8187710) [38]. Association of *ABCC2* rs3740066 with cardiac parameters was previously not published in the literature. We found the same gene but different *ABCC2* SNP to be associated with acute and chronic ACT. A possible explanation for this divergence might be the different phenotyping method, different target SNPs and the different population: age groups, tumor types, and chemotherapies. Armenian et al. revealed that the rare allele of *ABCC2* rs8187710 was over-represented in survivors of hematopoietic cell transplantation patients who developed anthracycline-related congestive heart failure [71]. A meta-analysis of twenty-eight studies found increased risk of ACT in a strong association within *ABCC2* gene, with the above mentioned rs8187710 SNP, which is near to rs3740066 [50]. There have also been several studies investigating *ABCC2* rs3740066. A research of Lopez-Lopez et al. studied the methotrexate (MTX) plasma levels and SNPs in pediatric ALL patients, focusing on adverse events. They suggest rs3740066 as a predictor to prevent MTX toxicity [9]. Hegyi et al. investigated the pharmacokinetics of MTX among osteosarcoma pediatric patients. In their analysis AUC_0–48_ (area under the concentration–time curve) was significantly lower in patients with homozygous variant genotype of rs3740066 [52]. The potential function of rs3740066 SNP is not fully understood yet. It may modify the mRNA stability or act together with rs572344237 SNP at the transcriptional level [72].

### NQO1

In our study, rs1043470 was connected with reduced cardiac function rates during the treatment and between the fifth and tenth years after the therapy. Rs1043470 is located in the 3’UTR region of both *NQO1* (nicotinamide adenine dinucleotide phosphate: quinone oxidoreductase 1) and *NFAT5* (Nuclear Factor Of Activated T-Cells 5) genes, as *NQO1* is transcribed from the complementary strand. Nuclear factor-activated T- cell 5 (NFAT5) plays a role against hyperosmotic stress, it is also expressed in the heart. NQO1 is a cytoplasmic 2-electron reductase, it reduces quinone to hydroquinone. NQO1 prevents oxidative stress and defends against pro-oxidant drugs like anthracyclines [73]. The SNP which seemed to be relevant in our study has not been studied in the literature yet, although there are several SNPs in the *NQO1* gene which were reported to be important from a clinical point of view. In a study of childhood ALL patients the outcome was worse in carriers of an *NQO1* variant [74]. Dunna et al. studied the effect of rs1800566, which is only approximately 7000 base pair distance away from the rs1043470 investigated in our study. Rs1800566 (NQO1*2) was associated with poorer outcome in patients treated with anthracycline for breast cancer [75]. Szkandera et al. in a breast cancer population failed to demonstrate the effect of rs1800566 on the therapy-response of anthracyclines [76]. In a cardiomyocyte cell culture investigation of *NFAT5* showed lower protein levels, but not on the mRNA level after doxorubicin treatment. Effects of doxorubicin were the degradation of NFAT5 protein and limitation of the viability of cardiomyocytes. Ito et al. proposed NFAT5 as a new positive marker of cardiomyocyte survival [77]. Lagoa et al. studied rats treated with doxorubicin. They experienced the down-regulation of *Nqo1* and increasing ROS production during the therapy. They suggested using this molecule as an early biomarker in the doxorubicin cardiotoxicity [78].

Rs1043470, studied by us, is located in a 3’UTR region of both *NQO1* and *NFAT5*. The localization of the SNPs might provide new binding sites for miRs (microRNAs) or affect the binding ability of them and these may result changes in the translation. According to the PolymiRTS Database 3.0 database, one miR binds our investigated SNP, it is called hsa-mir-6863 [79]. However, presently it is not known whether *NQO1*, *NFAT5* or both have a role in this respect.

### *SLC22A6* and *SLC28A3*

According to our results five to ten years after the diagnosis rs6591722 of *SLC22A6* gene was in correlation with lower cardiac function. This is the first time that *SLC22A6* gene polymorphism is found to be associated with cardiac function. Solute Carrier Family 22 Member 6 is involved in renal excretion of organic anions, toxic ones are also included. Renal *Slc22a6* was down-regulated after MTX treatment in rats [80]. In an in vitro study, indoxyl sulfate correlated adverse cardiac effects were inhibited by *SL22A6*, it blocked entering the toxin into cardiac cells [81].

The genotype distribution of the *SLC28A3* rs7853758 was also significantly different between cases and controls. In many studies *SLC28A3* rs7853758 is proved to be a very important protective genetic marker against ACT, its minor allele (A) found more often in controls than in patient cases [37, 82]. This SNP was recommended for clinical use in pharmacogenetic testing before using doxorubicin or daunorubicin in pediatric cancer patients’ treatment. [49]. This correlation for chronic cardiotoxicity was not significant in another cohort [83], nor RICOVER-60 trial found association with adverse cardiac reactions [84]. In contrast to these analyses, in our cohort the *SLC28A3* rs7853758 AA genotype was more frequent among cases. However, not only in the case- control study but also in the BN-BMLA we could confirm the importance of this variant which needs further validation in larger cohorts.

### CYP3A5

The *CYP3A5* rs4646450 TT genotype associated with fractional shortening lower than 28% in our joined cohort. The BN-BMLA showed that the *CYP3A5* rs776746 was potentially relevant in our case-control analysis. Previous studies in the literature did not find association of *CYP3A5* rs4646450 with cardiac parameters [48]. *CYP3A5* rs776746 AG/AA seemed to increase the risk of grade 2–4 cardiac toxicity in diffuse large B-cell lymphoma patients [85]. Our BN-BMLA analysis revealed potential strongly relevant effect of rs776746 SNP in gene CYP3A5 with respect to the case-control status of the patients. *CYP3A5* gene (7q21.1) is a member of cytochrome P450 proteins involved in drug metabolism, synthesis of steroids and lipids. CYP3A5 is expressed in the liver and also in extrahepatic tissues e.g. in intestines. Genetic variability of *CYP3A5* is high; it is not expressed in 20% of African and 80% of Caucasian population. SNPs in *CYP3A5* (rs776746 and rs10264272) may modify its alternative splicing and protein truncation, which can result in a less active CYP3A5 [86]. Huang et al. studied the CYP3A5 enzyme activity with the presence of its different gene polymorphisms in pediatric ALL patients. They revealed that patients with rs776746 had lower enzyme activity. Rs776746 was in association with the mRNA expression, daunorubicin plasma concentration and adverse drug reactions. In this investigation the AUC of daunorubicin was higher in children with cardiotoxicity [87]. In our population the intronic *CYP3A5* rs4646450 SNP, was associated with low FS (< 28%, cases) with a significantly higher OR value in males indicating gender related differences. Both male and female gender has been already shown as risk factors for developing cardiotoxicity [88–90]. Difference in the adverse drug reactions between man and woman might be explained with their genetic background and there might be associations only in one of the genders [91]. Presently, the function of this SNP is not known, however, in a study rs4646450 was in correlation with reduced protein-expression and activity of *CYP3A4* in human liver [92]. However, these results require further validation.

## Conclusions

In this study we confirmed that genetic variations in genes coding transporters and metabolizing enzymes might influence the anthracycline-induced cardiotoxicity. For the utilization of these findings, further validations and functional analysis of the implicated genetic variations are needed. International cooperation is required to be able to gather patient population with appropriate statistical power. The confirmed variants should be tested in clinical trials with long-term follow-up, because of late cardiotoxicity. Today we are only at the beginning of this process, but regarding the huge development of medicine in the last few decades and with the help of a user-friendly decision–support system, we can be sure that the number of usable pharmacogenomic tests will be expanded in the future, contributing to more effective personal therapies.

## Additional file

Additional file 1: Information about the studied SNPs. Information on the selected SNPs is shown in an additional table file in more detail (gene, SNP ID, chromosome, position, function, alleles and minor allele frequencies). (XLSX 14 kb)

## Abbreviations

ABCB1: ATP Binding Cassette Subfamily B Member 1
ABCC1: ATP Binding Cassette Subfamily C Member 1
ABCC2: ATP Binding Cassette Subfamily C Member 2
ACT: anthracycline-induced cardiotoxicity
ALL BFM: Berlin–Frankfurt–Münster
ALL: acute lymphoblastic leukemia
AUC: area under the concentration–time curve
BN-BMLA: Bayesian network based Bayesian multilevel analysis of relevance
COSS: German-Austrian-Swiss osteosarcoma study group
CYP3A4: Cytochrome P450 Family 3 Subfamily A Member 4
CYP3A5: Cytochrome P450 Family 3 Subfamily A Member 5
ECHO: echocardiography
EF: ejection fraction
FS: fractional shortening
GWA: genome-wide association
LVEDD: Left ventricular end-diastolic-diameter
LVESD: Left ventricular end-systolic-diameter
miR: microRNA
MTX: methotrexate
NFAT5: Nuclear Factor Of Activated T-Cells 5
NQO1: NAD(P)H Quinone Dehydrogenase 1
OR: odds ratio
OSC: osteosarcoma
RARG: Retinoic Acid Receptor Gamma
SLC22A6: Solute Carrier Family 22 Member 6
SLC28A3: Solute Carrier Family 28 Member 3
SNP: single nucleotide polymorphism
VACTERL: vertebral defects, anal atresia, cardiac defects, tracheo-esophageal fistula, renal anomalies, and limb abnormalities
